# High-Throughput Characterization of Human Fibroblast Responses to a Panel of *Staphylococcus aureus* Antigen Proteins Using Olink Proteomics

**DOI:** 10.1093/ofid/ofag545

**Published:** 2026-09-02

**Authors:** Rajaa S D Dalloul, Muhammad U Sohail, Sareena Chennakkandathil, Hina Sawarth, Muna Al-Noubi, Sunkyu Choi, Frank Schmidt

**Affiliations:** Weill Cornell Medicine Qatar, Proteomics Core, Doha, Qatar; Weill Cornell Medicine Qatar, Proteomics Core, Doha, Qatar; PremieHealth Pty Ltd., Jumar Bioincubator, Melbourne, VIC, Australia; Weill Cornell Medicine Qatar, Proteomics Core, Doha, Qatar; Weill Cornell Medicine Qatar, Proteomics Core, Doha, Qatar; Weill Cornell Medicine Qatar, Proteomics Core, Doha, Qatar; Weill Cornell Medicine Qatar, Proteomics Core, Doha, Qatar; Weill Cornell Medicine Qatar, Proteomics Core, Doha, Qatar

**Keywords:** antigen proteins, immune response markers, inflammatory cytokines, Olink proteomics, *Staphylococcus aureus*

## Abstract

**Background:**

*Staphylococcus aureus* is a major human pathogen that can elicit immune-inflammatory responses and infections, largely driven by its broad repertoire of antigenic proteins. Understanding these factors is valuable for elucidating mechanisms of infection.

**Method:**

Fifty-two recombinant *S. aureus* antigen proteins were individually applied to cultured human dermal fibroblasts (HDFs). Following antigen stimulation of confluent HDFs (52 antigens + 44 controls; in 3 replicate plates), host protein responses were quantified using Olink inflammation, cardiovascular II, and III proteomic platforms. Data were analyzed using unsupervised clustering, differential expression analysis, and correlation network modeling to identify patterns of immune-inflammatory signals.

**Results:**

Unsupervised analysis identified 3 distinct host-response clusters, with concordant separation observed across hierarchical clustering (Ward.D2) and t-distributed stochastic neighbor embedding (t-SNE) projection. These clusters can be broadly defined as a cytotoxic/inflammatory antigen cluster (Cluster_1), enriched for key virulence factors (eg, HlgA, Atl.1, SasG.2) and characterized by marked upregulation of inflammatory mediators (including IL-6, IL-8, CXCL1, and CCL3). An immune modulation/surface protein cluster (Cluster_2), comprising adhesins and immune-evasion proteins (eg, Chp, SSL11, Atl.2), was associated with selective upregulation of AXIN1, IL-33, and DECR1. A small outlier antigen cluster (Cluster_4), consisting of LuKE and SdrD.1, did not exhibit a clearly defined host-response profile. Differential expression analysis identified 12 core host proteins—including COL1A1, CXCL1, CXCL16, DLK-1, GLO1, IGFBP-1, IL33, IL6, IL8, MCP-1, TNF, and TRAIL-R2—with consistent and significant changes across clusters (false discovery rate [FDR]-adjusted *P* < .01).

**Conclusions:**

This preliminary study suggests that *S. aureus* antigen proteins trigger a coordinated immune-inflammatory cascade in HDFs.

## Teaser

Exposure of human dermal fibroblasts to 52 recombinant *S. aureus* antigens revealed distinct immune-inflammatory responses. Cytolytic toxins and leukocidins triggered coordinated pro-inflammatory cascades markedly upregulating IL-6, IL-8, and CXCL1, whereas immune-evasion proteins elicited selective, moderate responses, highlighting antigen-specific host responses to *S. aureus* virulence factors.

*Staphylococcus aureus* is one of the most common pathogens, capable of causing a wide spectrum of infections, ranging from asymptomatic colonization to severe local or systemic disease [1]. A major contributor to the pathogenic success of *S. aureus* is its extensive repertoire of virulence factors, including cytolytic antigens, immune-evasion proteins, adhesins, and enzymes [2, 3]. Many of these factors directly interact with host cells, triggering inflammatory signaling, cellular stress, and immune modulation [4, 5]. Classic examples include pore-forming toxins such as Hla and bicomponent leukocidins (eg, LukE/LukF), as well as γ-hemolysin complexes (HlgAB/HlgCB), which disrupt cellular membranes and activate inflammatory pathways, as well as superantigen-like proteins and complement inhibitors that manipulate host immune responses to promote bacterial survival [6–8].

Host responses to *S. aureus* antigens are often examined at the level of individual virulence factors. For example, Efb, SpA, and LukF can induce cytokine production, activate innate immune pathways, and trigger cell death [1, 9]. In line with this, Hla, which engages ADAM10 as its cellular receptor, has been shown to stimulate the production of inflammatory mediators, including IL-6, IL-8, and TNF, by activating epithelial and immune cells [10]. Moreover, many *S. aureus* leukocidins disrupt host defense mechanisms, including complement activation, neutrophil recruitment, and antibody-mediated responses [9]. While these studies have provided important mechanistic insights, they have largely focused on individual antigens or small sets of virulence factors.

Despite the large number of characterized *S. aureus* antigens, a comprehensive comparison of host inflammatory responses induced by a broad panel of virulence-associated proteins has been lacking. While previous studies have characterized humoral responses to a broad range of *S. aureus* antigens, focusing on IgG and IgA profiles in the general population [2], these approaches primarily reflect exposure and immunogenicity rather than the functional impact of antigens on host cellular signaling and downstream inflammatory responses. It remains unclear whether different antigens trigger similar host-response programs or whether distinct virulence factors induce specific inflammatory signatures. A systematic comparison of antigen-induced host responses may therefore help to reveal functional relationships between antigens and identify groups of virulence factors that converge on shared host signaling pathways.

Recent advances in multiplex proteomic technologies allow simultaneous quantification of large numbers of secreted host proteins, providing an opportunity to investigate antigen-induced inflammatory responses at a systems level. The Olink proximity extension assay platform enables sensitive and high-throughput measurement of cytokines, chemokines, and other immune mediators in biological samples, making it well-suited for profiling host inflammatory responses in vitro.

Previously, we used mass spectrometry and immunoproteomics to characterize *S. aureus* antigen proteins from nasal polyp tissues [11, 12]. Building on this work, we here extend our analysis to host inflammatory responses induced by these antigens. As we and others have previously reported, *S. aureus* is one of the leading causes of skin and soft tissue infections [13, 14]. Because dermal fibroblasts are key resident cells involved in tissue homeostasis, innate immune responses, and wound repair during these infections, we selected primary human dermal fibroblasts (HDFs) as our experimental model. HDFs were exposed to individual antigens, and the resulting secretion profiles of host proteins were quantified. Using unsupervised clustering, correlation network analysis, and differential expression approaches, we compared the host-response signatures triggered by different virulence factors. This approach allowed us to identify groups of *S. aureus* antigens that induce similar inflammatory programs and to characterize the host-response pathways associated with these antigen clusters.

## METHODS

### Cell Lines and Culture

Cryopreserved HDFs were obtained from Lonza Cell Bio Services (Cat# CC-2511). The vial contained ≥5 × 10^5^ cells, stored in vapor-phase liquid nitrogen. The cells were rapidly thawed in a 37°C water bath with gentle agitation, ensuring that vial caps were not submerged. Once thawed, vials were disinfected with 70% ethanol and transferred to a biosafety cabinet. Cells were immediately diluted into 10 mL of pre-warmed Dulbecco's Modified Eagle Medium (DMEM) (Thermo Fisher, Cat# 11965092) and centrifuged at 300 × *g* for 10 minutes at 20°C. The supernatant was aspirated, and the cell pellet was resuspended in fresh DMEM for cell counting using a hemocytometer. The cells were seeded at a density of 0.5 × 10^5^ cells per T-75 flask and incubated at 37°C in a humidified atmosphere containing 5% CO_2_. Culture medium was replaced every 2 days until cells reached 80%–90% confluence (typically within 3–5 days).

For passaging, cells were washed 3–4 times with sterile phosphate-buffered saline (PBS) to remove residual cell culture. Cells were trypsinized with 2 mL of 0.25% trypsin–2.21 mM ethylenediaminetetraacetic acid (EDTA) (Sigma-Aldrich, Cat# T4049) per T-75 flask for 5 minutes at 37°C. Trypsin was neutralized with DMEM, and complete detachment was verified under a microscope. Cells were collected, counted, and seeded at 5 × 10^3^ cells per well in 96-well plates and cultured to ∼80% confluence.

### *S. aureus* Antigen Stimulation

A panel of 52 recombinant *S. aureus* virulence factors, encompassing toxins, enzymes, and surface proteins, was kindly provided by Prof. Uwe Völker and colleagues (Institute of Genetics and Functional Genome Research, University Medicine Greifswald, Germany). The recombinant proteins used in this study were generated, purified, and quality-controlled as described previously [2]. Each factor was applied individually to cells overnight at a standardized concentration of 1 μg per well (n = 52 wells), while untreated control cells served as controls (n = 44 wells). Each antigen was applied individually at a standardized concentration of 1 μg/ml, as no consensus working concentration exists across the diverse *S. aureus* virulence factors included in this study, thereby enabling direct comparison of host responses under identical experimental conditions. Cell morphology was monitored by microscopy before and after antigen treatment to detect any overt morphological changes or extensive cell detachment indicative of cytotoxicity. Supernatants from 3 replicated treatments and control experiments (10 μL aliquots) were analyzed using Olink Target proteomics panels (Inflammation and CVD II and III).

### Olink Proteomic Analysis

For affinity-based analysis, cell culture supernatants from 96 samples, 52 of which were treated with bacterial protein antigens and 44 controls, were analyzed using the Olink Target 96 platform (Olink, Uppsala, Sweden). The Olink assays used included Inflammation (Olink, Cat# 3025), CVD II (Olink, Cat# 5007), and CVD III (Olink, Cat# 6114). Protein levels in cell culture supernatants were quantified using Proximity Extension Assay technology [15]. Proteins represented on multiple Olink panels were distinguished by appending the suffixes “_1” and “_2” to the protein labels throughout all figures and analyses.

### Real-time PCR

High-throughput real-time PCR of reporter DNA linked to protein-specific antibodies was performed on a 96-well Fluidigm integrated chip (Fluidigm, San Francisco, CA), with signal detection on a Biomark HD system. Cycle threshold (CT) values were extracted from raw data using Fluidigm RT-PCR software and subsequently processed with Olink normalized protein eXpression (NPX) Manager. The log2-transformed CT values were internally normalized using spiked-in controls, inverted to obtain NPX values, and adjusted for inter-plate control medians and intensity scaling across all samples and plates. Final NPX values were further normalized to the median using Genedata Analyst (Basel, Switzerland, v.1.6.0.0).

### Data Analysis

Olink proteomic data were analyzed using RStudio (2026.04.0+526). Unsupervised hierarchical clustering was performed using Ward's minimum variance method (Ward.D2) on scaled NPX values to identify host-response profiles across *S. aureus* protein treatments. Dimensionality reduction was performed using principal component analysis (PCA), followed by t-distributed stochastic neighbor embedding (t-SNE) to visualize sample clustering in a low-dimensional space. The Ward.D2 and t-SNE algorithms effectively organized the samples into 4 distinct clusters: 3 representing treatment groups and 1 representing the control.

Data were visualized using heatmaps with corresponding dendrograms to assess sample similarity. Differential abundance analysis between antigen-treated samples and controls was performed using volcano plots that were generated to visualize Olink proteins with significantly altered expression levels based on effect size and statistical significance. Correlation analyses were conducted using Pearson correlation coefficients to identify co-regulated proteins. All data visualization and statistical analyses were performed using R packages, including shiny, tidyverse, pheatmap, ggplot2, corrplot, and Rtsne.

### Patient Consent

The work was performed on commercial cell line and *S. aureus* protein antigens. Therefore, patient consent was not required.

## RESULTS

### Global Response Profiling of Antigen Proteins

**Figure 1.**
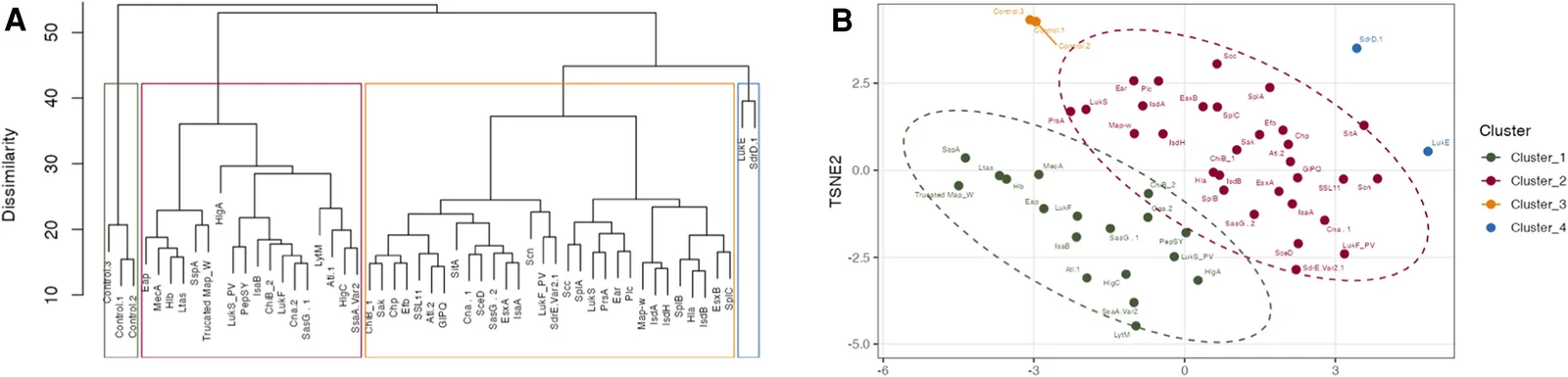
Unbiased clustering reveals distinct groups of *S. aureus* proteins inducing similar host inflammatory responses. *A*, Hierarchical clustering of *S. aureus* proteins based on their induced host protein secretion profiles measured with the Olink panels. Clustering was performed using Ward's method (Ward.D2). The dendrogram grouped bacterial proteins according to the similarity of the host inflammatory signatures they induce. *B*, t-distributed stochastic neighbor embedding (t-SNE) visualization of the same dataset based on principal component–reduced host-response profiles. Each point represents one *S. aureus* protein, positioned according to similarity in the host inflammatory response pattern. Colors denote clusters identified from the hierarchical clustering analysis.

To investigate the overall host response to *S. aureus* antigen proteins, unsupervised clustering analyses were performed on Olink-derived NPX profiles. Hierarchical clustering using Ward's method revealed clear segregation of samples into multiple distinct clusters based on similarity in host-response signatures (Figure 1*A*). This segregation indicates that individual *S. aureus* antigens elicit reproducible and distinct host-response patterns rather than a uniform inflammatory response.

t-SNE visualization of PCA-reduced data demonstrated well-separated groups of *S. aureus* proteins in low-dimensional space (Figure 1*B*). Cluster_1 comprised predominantly cytolytic toxins and leukocidins (eg, HlgA, HlgC, LukD, LukS-PV), which were associated with relatively strong immune-inflammatory induction (Table 1). Cluster_2 included a mixture of immune-modulatory and surface-associated proteins (eg, Chp, SSL11, SasG), generally associated with a moderate and more selective host response. Cluster_3 represented the control group, characterized by minimal or baseline levels of inflammatory protein expression. In contrast, Cluster_4 contained a smaller subset of proteins (LukE and SdrD.1) that elicited distinct but comparatively weaker inflammatory signatures relative to Cluster_1.

**Table 1.** Host-Response Programs Associated With Dendrogram-Defined *S. aureus* Antigen Clusters

Toxin Cluster (Based on Host-Response Clustering)	Toxin Members	Dominant Toxin Functional Class	Top Distinguishing Olink Proteins	Host-Response Program	Biological Interpretation	Potential Relevance for Antibody Targeting
Control cluster	Control 1, Control 2, Control 3	No toxin exposure	COL1A1, THBS2, SPON2	Baseline tissue homeostasis	Reflects unstimulated cellular state with extracellular matrix and structural protein signaling dominating in the absence of toxin-induced inflammation	Serves as reference baseline
Cytotoxic/inflammatory toxin cluster	HlgA, Hlb, LukS-PV, LukF-PV, ChiB variants, SasG.2, SsaA.Var2, Atl.1, MecA, Truncated Map_W	Cytolysins, leukocidins, membrane-active toxins	IL8, CXCL1, CXCL5, IL6, CCL3, CCL4, IL1α, CXCL6	Acute inflammatory chemokine storm	Strong activation of neutrophil-recruiting chemokines and pro-inflammatory cytokines, consistent with membrane damage, danger signaling, and acute innate immune activation	These toxins strongly stimulate inflammatory signaling and may represent immunodominant targets for neutralizing antibodies
Immune modulation/surface protein cluster	Chp, SSL11, Atl.2, Cna.1, SceD, SasG.1, IsaA, Scn, SdrE.Var2.1, SplA, LukS, PrsA, Ear, Efb, Map-W, IsdH, SplB, IsaB, EsxB	Adhesins, immune-evasion factors, surface proteins, proteases	AXIN1, IL33, DECR1, moderate IL6, CXCL5, CCL3	Stress and immune-modulatory response	These proteins induce a mixed host response characterized by moderate inflammatory signaling combined with cellular stress and remodeling pathways rather than maximal chemokine induction	Such proteins may shape host-pathogen interaction without causing strong cytotoxicity and could represent antigenic targets recognized during natural infection
Small outlier toxin cluster	LukE, SdrD.1	Leukotoxin/adhesion-related factor	Not statistically resolved	Distinct but weak response signature	The separation of this cluster suggests a host-response program distinct from the larger toxin groups, although statistical power is limited due to small cluster size	Requires further investigation to determine biological relevance

The table summarizes the relationship between antigen clusters identified through host-response clustering and the corresponding biological response programs observed in the Olink Inflammation dataset.

### Olink Protein-Level Analysis

**Figure 2.**
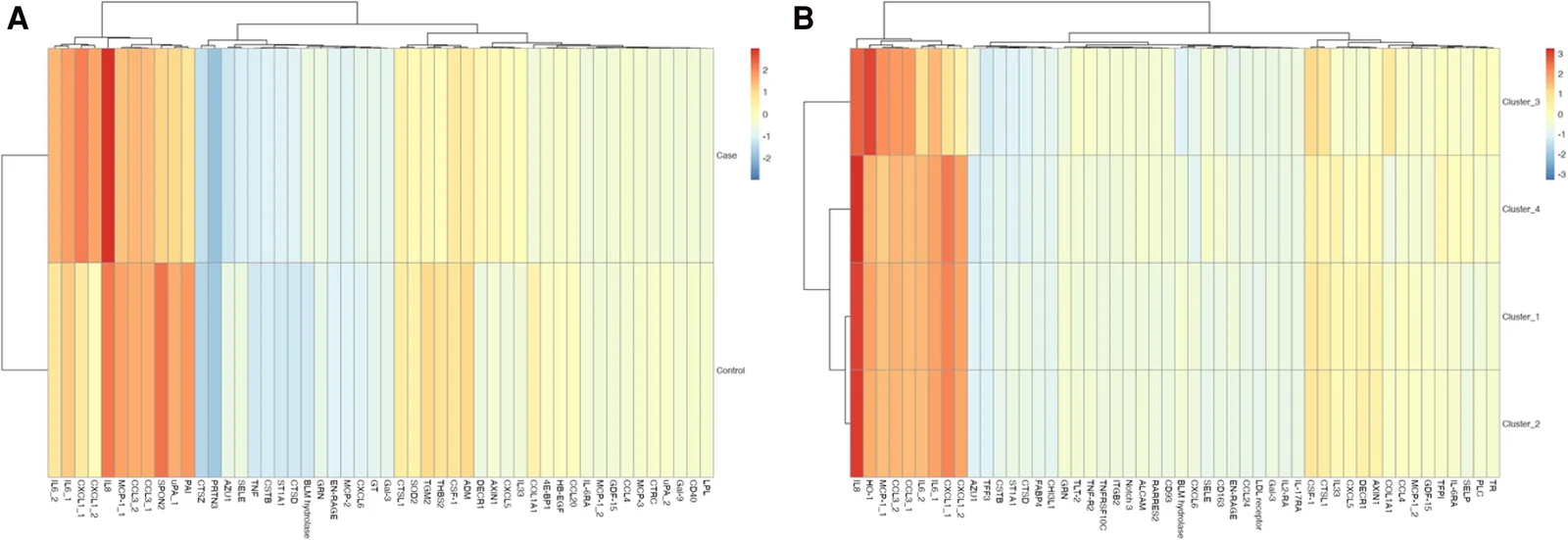
Differential host inflammatory signatures induced by *S. aureus* proteins. *A*, Heatmap showing the 50 most strongly regulated human proteins measured by the Olink panels when comparing all *S. aureus* protein stimulations collectively against medium control conditions. *B*, Heatmap displaying the 50 most differentially regulated host proteins identified when comparing the antigen clusters defined in the unbiased clustering analysis.

Heatmap visualization of the most strongly regulated proteins revealed clear differences in expression profiles between *S. aureus* antigen-treated and control samples (Figure 2*A*) and between antigen-treated clusters (Figure 2*B*). Figure 2*A* presents a heatmap of the top 50 significantly regulated human proteins, showing clear differential expression between treatment and control groups (FDR-adjusted *P* ≤ .05, Benjamini-Hochberg [BH]). Among these, key discriminating markers include IL6, CXCL1, IL8, MCP-1, CCL3, SPON2, UPA, and PAI (Z-score ≥ 2). Notably, IL6, CXCL1, and IL8 are elevated in the treatment group, whereas SPON2, UPA, and PAI show higher expression in controls. Similarly, Figure 2*B* presents a heatmap of the top 50 significantly regulated human proteins across 4 clusters, comprising *S. aureus* antigen–treated (Clusters_1, _2, and _4) and controls (Cluster_3). Distinct expression patterns are evident across clusters, underscoring heterogeneity in host-response profiles. Certain clusters exhibit elevated levels (FDR-adjusted *P* ≤ .05, BH) of pro-inflammatory markers, including IL6, IL8, and CXCL1. In contrast, others show comparatively reduced expression of proteins, such as HO-1, MCP1, and CCL3, or maintain baseline levels of IL2RA, IL17RA, CXCL6, and CCL24. These findings are further supported by individual *S. aureus* protein treatment heatmaps, which consistently reflect the cluster patterns identified above (Supplementary Figure 1). These patterns highlight functional differences in the biological activity of the corresponding *S. aureus* protein clusters. Importantly, these cluster-specific signatures were consistent with the global separation observed in the unsupervised analyses (Figure 2), supporting the robustness of the identified response groups. Furthermore, mirroring the clustering observed for *S. aureus* antigens, hierarchical clustering of Olink protein responses using Ward's D2 linkage identified 4 distinct clusters (Supplementary Figure 3A). This clustering was further supported by t-SNE visualization of principal component–reduced host-response profiles (PCluster_1–4).

**Figure 3.**
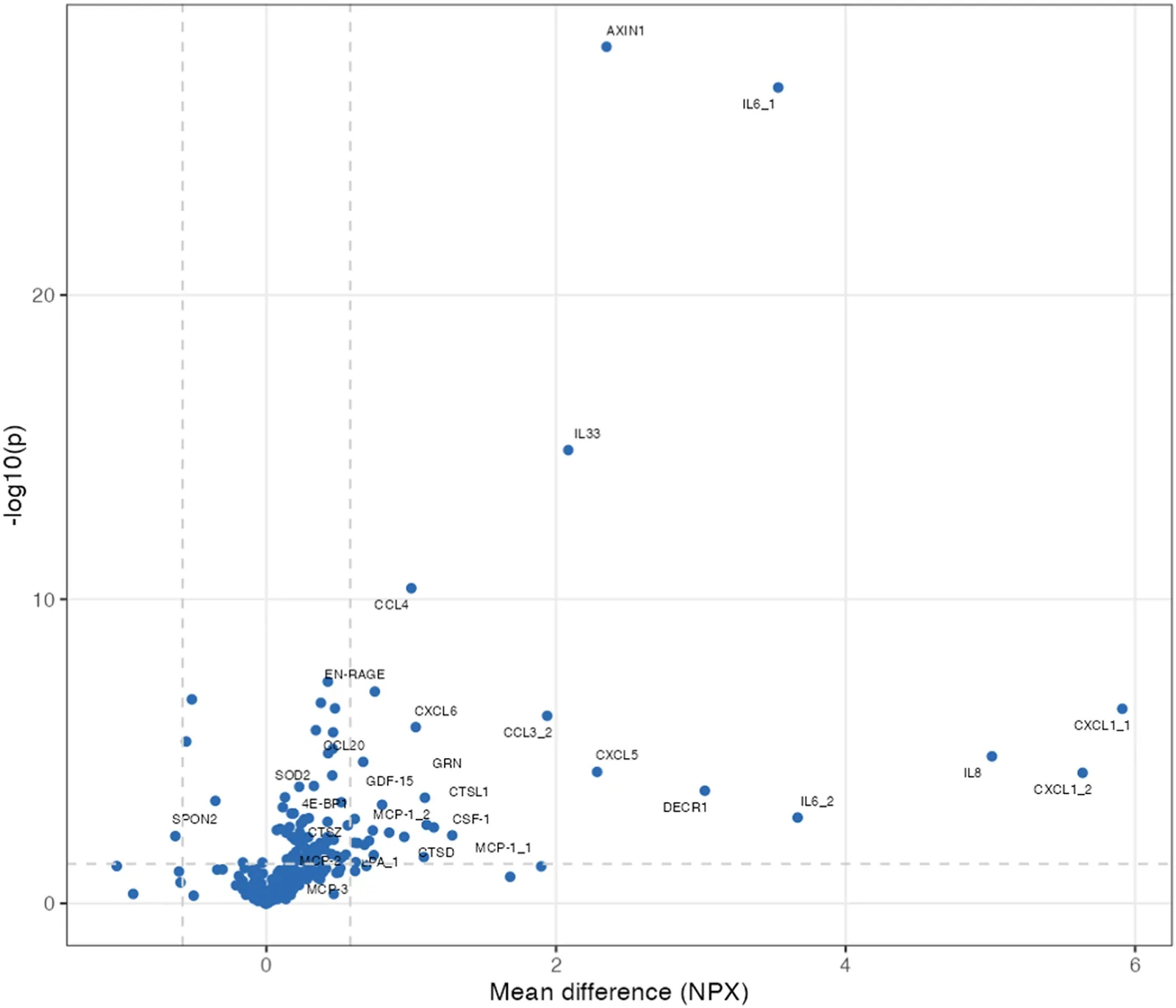
Volcano plot identifying host proteins significantly altered in response to *Staphylococcus aureus* antigen exposure. The volcano plot summarizes the differential abundance of human proteins measured with the Olink Inflammation, CVDII, and CVDIII panels when comparing all antigen-treated samples to medium controls. The x-axis shows the mean difference in NPX between antigen-treated and control clusters, while the y-axis displays the statistical significance as −log10 (*P*-value) derived from Welch's t-tests. *P*-values were corrected for multiple testing using the Benjamini–Hochberg procedure.

Figure 3 and S2 present volcano plots summarizing differential host protein responses (ΔNPX; log2 fold change) following *S. aureus* antigen treatment, comparing different clusters. Among the most strongly upregulated proteins, CXCL1, IL6, and IL8 showed the highest positive mean differences (FDR-adjusted *P* ≤ .05, BH). Other notably elevated proteins included AXIN1, IL33, CCL4, CXCL5, and DECR1, which also demonstrated substantial increases in abundance and statistical significance. When comparing Control with Cluster_1 and Cluster_2, volcano plots (Supplementary Figures 2A and 2B) revealed a broadly similar pattern of fold change and statistical significance across Olink-measured proteins, albeit with a notably different magnitude of response in Cluster_1. Direct comparison of Cluster_1 versus Cluster_2 identified a pronounced upregulation of key inflammatory mediators, including HO-1, HSP27, IL-8, CXCL1, IL-6, and CCL3, in Cluster_1, indicative of a robust acute inflammatory response characterized by enhanced neutrophil recruitment and activation of innate immune pathways (Supplementary Figure 2C).

**Figure 4.**
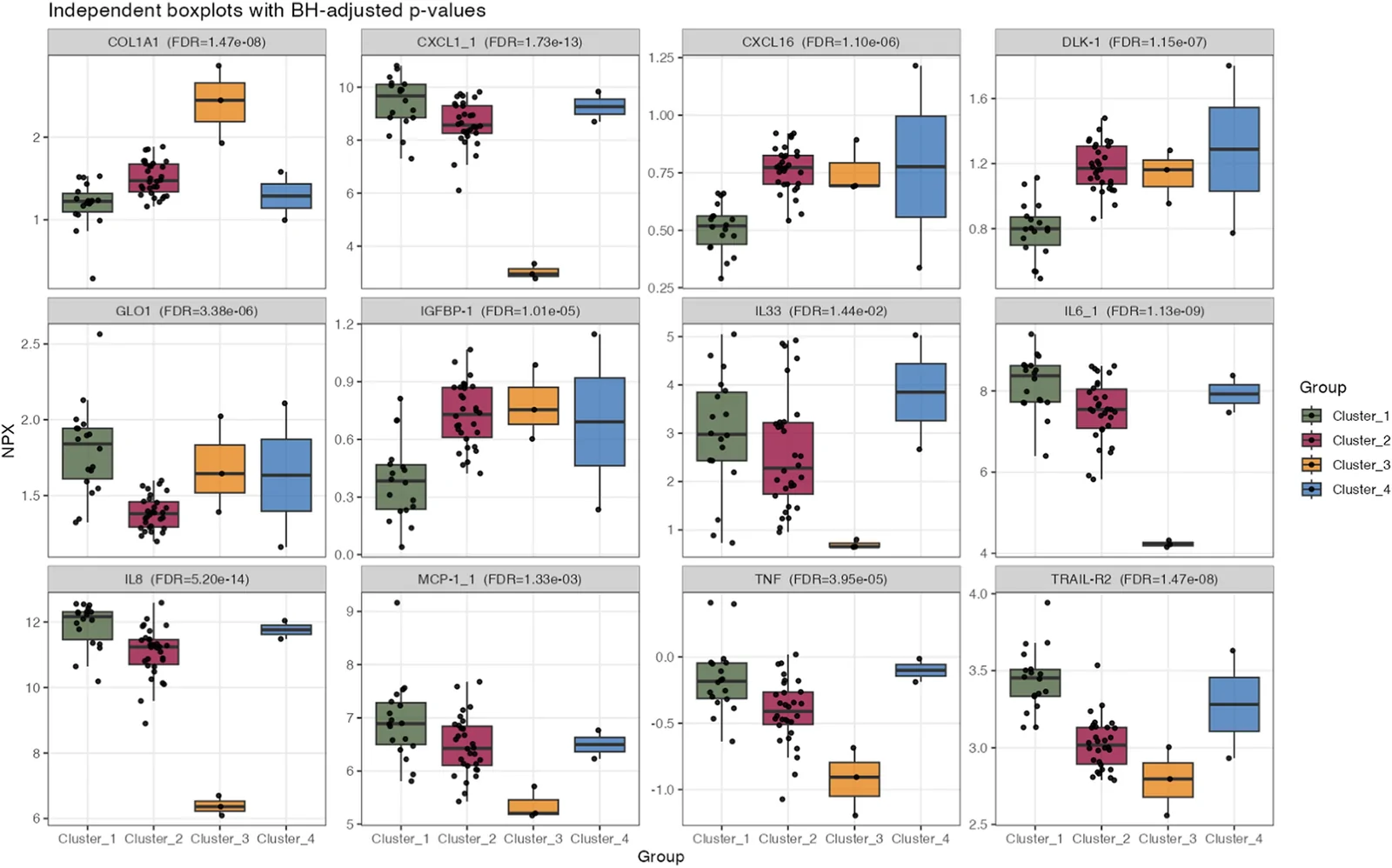
Independent differential analysis of the 12 most strongly regulated host proteins across the 4 cluster groups. Boxplots of the 12 host proteins with the strongest differential regulation across the 4 independently defined clusters. Protein abundance is displayed as NPX measured using the Olink Inflammation, CVDII, and CVDIII panels. Each panel represents one host protein, and each boxplot shows the distribution of NPX values between clusters 1 and 4. Statistical significance was assessed by independent comparisons with Benjamini–Hochberg (BH) correction for multiple testing, and the corresponding false discovery rates (FDR) are indicated above each panel.

Building on the global profiling (Figure 2) and differential analysis (Figure 3), the most significantly altered Olink proteins identified across these analyses were further subjected to cluster-level comparison. Figure 4 presents an independent differential analysis of the 12 top-regulated proteins across the 4 clusters. Consistent with earlier findings, key inflammatory mediators such as CXCL1, IL6, IL8, CXCL16, MCP-1, TNF, and IL33 show statistically significant differences between the clusters (BH-adjusted FDR ≤ 0.05), reinforcing the presence of heterogeneous host-response states. In addition, proteins associated with tissue remodeling and stress responses, including COL1A1, GLO1, IGFBP1, DLK1, and TRAIL-R2, also vary across clusters, further supporting functional stratification of *S. aureus*–induced host responses.

### Correlation Network of Olink Proteins

**Figure 5.**
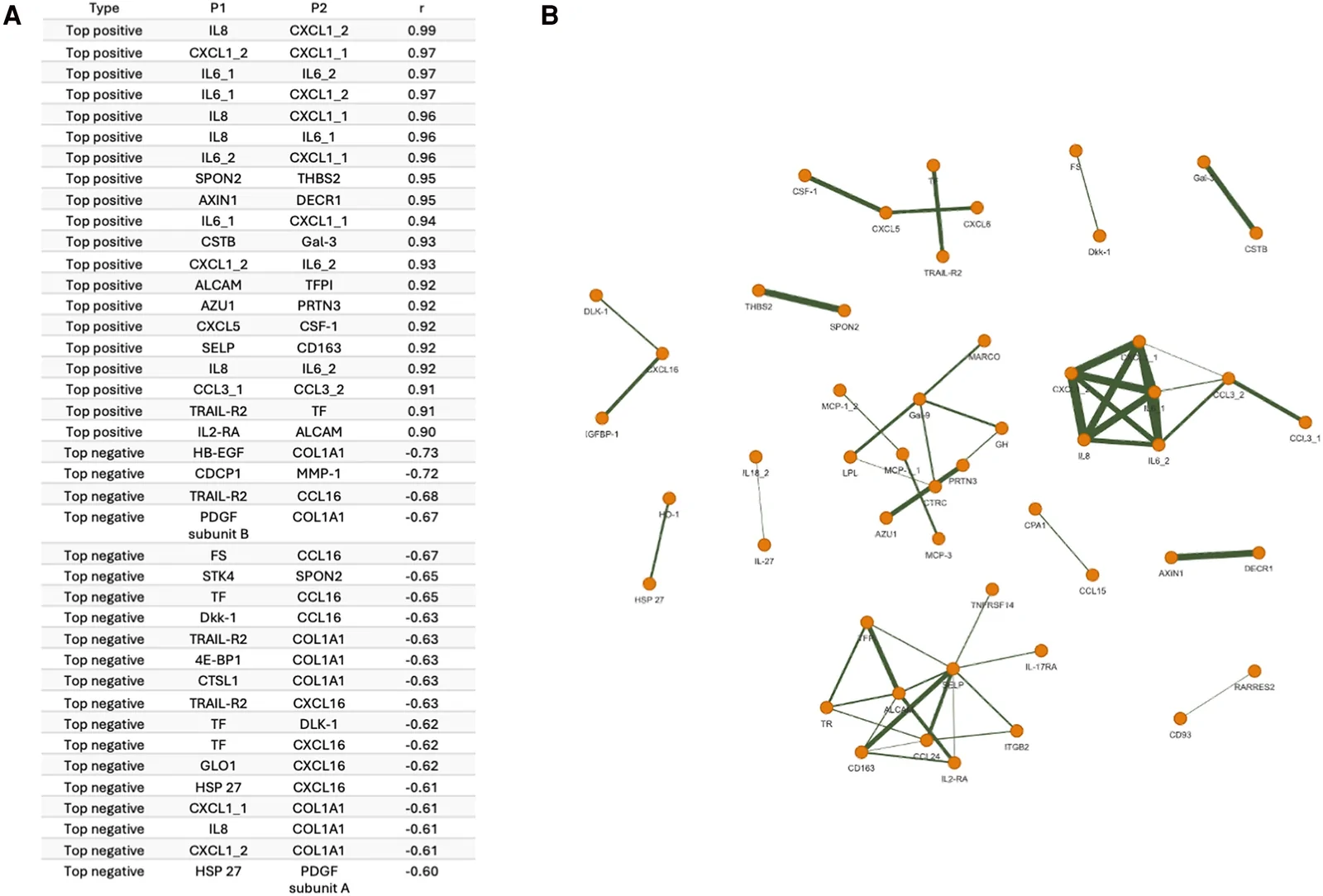
Correlation network of Olink-measured host proteins reveals coordinated inflammatory modules. *A*, The table summarizes the strongest positive and negative correlations observed between host proteins measured across all antigen-stimulation experiments. Correlation coefficients (r) were calculated based on NPX across all clusters. The strong correlation indicates proteins that consistently increase or decrease together, suggesting shared regulatory pathways or coordinated biological responses. *B*, The figure shows a correlation network constructed from the Olink Inflammation, CVDII, and CVDIII panel data, including only strong associations between host proteins (Pearson correlation >0.85). Each node represents a measured human protein, and edges indicate highly correlated expression patterns across the antigen-stimulation experiments. The network, therefore, highlights groups of host proteins that behave similarly across the entire dataset and likely participate in related biological pathways.

Many of the regulated proteins show strong positive or negative correlations across antigen-treated samples. Correlation coefficients (r > 0.85) were calculated based on NPX values across all clusters (Figure 5). In Figure 5, each node represents a protein, and edges indicate highly correlated expression patterns across *S. aureus* antigen treatments. The analysis revealed distinct modules, with a prominent cluster of pro-inflammatory cytokines and chemokines (eg, IL8, CXCL1, IL6), alongside smaller subnetworks related to immune signaling and tissue remodeling. The presence of highly interconnected proteins suggests coordinated regulation of inflammatory pathways rather than independent protein-level responses. The strongest positive correlations indicate proteins that consistently increase or decrease together, such as IL8, CXCL1, CXCL5, and IL6, which show extremely high correlations (r up to 0.99).

## DISCUSSION

*S. aureus* is a clinically important pathogen that produces a wide array of virulence factors, including numerous antigenic proteins. These secreted molecules play key roles in the pathogenesis of infections affecting multiple organ systems, including the skin and superficial tissue, respiratory, gastrointestinal, urinary, and reproductive tracts [8, 13, 16]. While individual antigens and surface proteins have been extensively studied, accumulating evidence suggests that host responses to *S. aureus* are not uniform but instead reflect a complex and coordinated interplay of multiple antigen-driven pathways [17]. For example, to cause a skin and superficial tissue infection, *S. aureus* employs a host of virulence factors, including cytolytic proteins, superantigenic factors, cell wall-anchored proteins, and molecules used for immune evasion [18]. Consistent with this complexity, for a systemic infection, *S. aureus* expresses surface-associated adhesins, including Cna, Eap, SasG, and fibrinogen-binding proteins (Efb, SdrD, SdrE), which mediate attachment to host tissues [19]. Immune evasion is facilitated by factors such as Chp, Scn, and SSL11, which interfere with neutrophil recruitment and complement activation. Finally, secreted antigens, including Hla, HlgA, leukocidins, and Plc, contribute to host cell damage and amplification of inflammatory responses [20]. Previously, we also functionally characterized a panel of *S. aureus* proteins derived from nasal polyps, identifying factors involved in tissue binding and invasion (Hla, HlgA, Ssl, Chp), nutrient acquisition (IsdA, IsdB, Efb), and several putative toxins, highlighting the coordinated contribution of multiple virulence-associated proteins to *S. aureus* pathogenesis.

Here, we observed that *S. aureus* antigen treatment of HDFs induced a structured and heterogeneous host-response landscape characterized by distinct inflammatory clusters, differential protein expression patterns, and coordinated correlation modules. By integrating unsupervised clustering and t-SNE visualization (Figure 1*A* and 1*B*), *S. aureus* proteins were divided into 3 clusters. Cluster_1 was enriched for cytolytic toxins and leukocidins, including HlgA, HlgC, LukF, and LukS-PV, alongside proteins involved in cell wall remodeling and host adhesion such as LytM, Cna.1, Eap, and SasG, suggesting a coordinated virulence phenotype marked by active host interaction, structural turnover, and inflammatory potential [8, 21–23]. Cytolytic toxins and leukocidins grouped into Cluster_1 are associated with stronger inflammatory induction. As reviewed by Tam and Torres [24], a broad range of *S. aureus* virulence factors, including leukocidins (eg, LukS-PV/LukF-PV) and hemolysins (Hlb, HlgA/B), can directly activate or prime innate immune pathways, thereby amplifying pro-inflammatory cytokine release and coordinating host inflammatory responses. Consistent with this, the marked upregulation of IL6, IL8, CXCL1, CXCL5, and CXCL6 in Cluster_1 reflects a pronounced acute inflammatory response (Table 1, Figure 2*B*), characterized by enhanced neutrophil recruitment and activation of innate immune pathways, resulting in a coordinated pro-inflammatory signaling cascade across antigen-treated groups [25, 26].

Unlike Cluster_1, Cluster_2 was characterized by a broader repertoire of immune-modulatory, surface-associated, and metabolic proteins, including Chp, SSL11, Scn, Efb, IsdA/B/H, and SdrE, as well as selected toxins such as Hla and LukS/LukF_PV. Many of these factors are known to interfere with host immune recognition and effector functions [27]. Jong et al [27] reported that Chp and Scn inhibit neutrophil chemotaxis and complement activation, respectively, whereas SSL11 modulates immune signaling using neutrophil extravasation, priming, chemotaxis, and activation of neutrophils. In addition, iron-regulated surface determinants (IsdA, IsdB, IsdH) and adhesion-associated proteins (Cna, SasG, SdrE) support bacterial persistence through host adaptation and nutrient acquisition rather than direct host cell damage [27, 28]. Notably, the surface exposure and immunogenicity of Isd proteins, particularly IsdB, have led to their investigation as vaccine candidates, although translation to clinical efficacy has remained challenging [29, 30]. These observations further support the role of Cluster_2 proteins in host interaction and immune modulation rather than overt cytotoxicity. Hence, the differential pathogenic potential, driven by cytolytic toxins in Cluster_1 versus a combination of enzymes, adhesins, and immune-modulatory proteins in Cluster_2, is reflected in the magnitude of the host response in our results. Herein, Cluster_1 elicited a more severe pro-inflammatory profile compared to the more moderate and selective response observed in Cluster_2 (Figure 3*B*). However, these comparative responses should be interpreted within the context of the experimental design, where recombinant antigens were assessed at a standardized concentration. While this approach enables systematic comparison across diverse *S. aureus* proteins, it may not fully reflect the physiological abundance, localization, or exposure levels of individual bacterial factors during infection.

Differential abundance analysis using a volcano plot (Figure 3) identified a core set of host proteins that were significantly induced following antigen exposure. Prominent among these were classical inflammatory mediators, including IL6, IL33, CXCL8 (IL-8), CXCL1, CXCL5, and CCL3/CCL4. This indicates activation of innate immune pathways and chemokine-driven recruitment of neutrophils [31–33]. From the differentially expressed host proteins identified in heatmaps and volcano plots, we presented 12 proteins, comparing their concentrations across different clusters (Figure 4). Among these proteins that differentiated the response clusters, COL1A1 was the only protein that showed higher abundance in the controls (Cluster_3) than in the antigen-treated clusters. COL1A1 differences likely reflect inflammation-associated remodeling of extracellular matrix homeostasis rather than a simple unidirectional change in collagen production, as antigen-mediated regulation of COL1A1 and related matrix pathways appears to be highly context-dependent [34, 35].

This interpretation is further supported by the correlation network analysis, which showed that strongly interconnected inflammatory mediators, including IL8, CXCL1, CXCL5, and IL6, exhibited highly coordinated expression patterns (Figure 5). These proteins, particularly IL8 and CXCL1, reflect their shared role in neutrophil recruitment and acute inflammatory signaling [15]. Notably, these inflammatory proteins showed consistent negative correlations with COL1A1 (r ≈ −0.6 to −0.7), supporting an inverse relationship between inflammatory activation and extracellular matrix maintenance and highlighting a coordinated shift from tissue homeostasis toward inflammation-driven remodeling.

## CONCLUSION

While the present study provides insights into antigen-specific inflammatory responses induced by *S. aureus* antigens, the findings should be interpreted within the context of an in vitro HDF model and require further validation in more complex biological systems. This study identifies a structured and heterogeneous host-response landscape characterized by distinct inflammatory clusters and coordinated protein interaction networks. Cytolytic toxins were associated with robust pro-inflammatory responses, whereas immune-modulatory protein clusters elicited more selective and controlled host activation. Together, these findings provide a systems-level framework for understanding antigen-specific host responses and highlight candidate antigens and pathways for further investigation in the context of *S. aureus* infection biology.

## Supplementary Material

ofag545_Supplementary_Data

## Supplementary Data

Supplementary materials are available at *Open Forum Infectious Diseases* online. Consisting of data provided by the authors to benefit the reader, the posted materials are not copyedited and are the sole responsibility of the authors, so questions or comments should be addressed to the corresponding author.
