# Supplementary material for: High-Throughput Characterization of Human Fibroblast Responses to a Panel of Staphylococcus aureus Antigen Proteins Using Olink Proteomics
Source: Open Forum Infect Dis. 2026 Sep 2;13(9):ofag545. doi: 10.1093/ofid/ofag545 (PMC13579471; doi:10.1093/ofid/ofag545)
Supplement: Supplementary file 1 [file ofag545_supplementary_data.docx]

Supplement file

Figure S1: **Differential host inflammatory signatures induced by *S. aureus* proteins.**

A

B

(A) Heatmap showing the top 100 proteins most strongly regulated human proteins measured by the Olink panels when comparing all *S. aureus* protein stimulations. Color intensity reflects NPX, with red indicating increased and blue indicating decreased levels relative to the dataset mean.

(B) Heatmap displaying the top 50 most regulated human proteins identified.

**Figure S2:** **Volcano plot identifying host proteins significantly altered in response to *Staphylococcus aureus* antigen exposure.**

B

A

Volcano *S. aureus* Cluster 3 (Control) vs Cluster_2

Volcano *S. aureus* Cluster 3 (Control) vs Cluster_1

Volcano *S. aureus* Cluster_1 vs Cluster_2

C

The volcano plots summarize the differential abundance of human proteins measured with the Olink Inflammation, CVDII, and CVDIII panels when comparing Control with Cluster_1 (A) Control vs Cluster_2 (B), and Cluster_1 vs Cluster_2 (C). The x-axis shows the mean difference in NPX between treatment groups, while the y-axis displays the statistical significance as –log10 (p-value) derived from Welch’s t-tests. P-values were corrected for multiple testing using the Benjamini–Hochberg procedure.

**Figure S3:** **Unbiased clustering reveals distinct Olink proteins clustering inducing similar host inflammatory responses.**

A

B

(A) Hierarchical clustering using Ward.D2 linkage identified four distinct Olink protein clusters based on co-abundance patterns. Clustering was performed using Ward’s method (Ward.D2). The dendrogram grouped Olink proteins according to the similarity of the host inflammatory signatures they induce.

(B) t-distributed stochastic neighbor embedding (t-SNE) visualization of the Olink proteins based on principal component–reduced host-response profiles. Each point represents one Olink protein, positioned according to similarity in the host inflammatory response pattern. Colors denote clusters identified from the hierarchical clustering of these four groups (PCluster_1–4) at the protein level.
